# Response to In-Home Virtual Reality Program for Chronic Lower Back Pain: A Randomized Sham-Controlled Effectiveness Trial in a Clinically Severe and Diverse Sample

**DOI:** 10.1016/j.mcpdig.2023.11.010

**Published:** 2023-12-26

**Authors:** Jesper Knoop, Syl Slatman, Bart Staal

**Affiliations:** Musculoskeletal Rehabilitation research group, HAN University of Applied Sciences, Nijmegen, Netherlands; Department of Health Sciences, Musculoskeletal Health, Vrije Universiteit, Amsterdam, the Netherlands; Musculoskeletal Rehabilitation research group, HAN University of Applied Sciences, Nijmegen, Netherlands

In the past decade, therapeutic virtual reality (VR) has been introduced as a promising health technology, based on a fast-growing amount of literature. Therapeutic VR could offer many therapeutic options in rehabilitation and other (health care) settings, with a number of suggested underlying working mechanisms, such as distraction, neuromodulation, graded exposure, and increased adherence.^1^, ^2^, ^3^ The randomized controlled trial (RCT) conducted by Maddox et al^4^ in the current issue of Mayo Clinic Proceeding: Digital Health is a valuable addition to the body of evidence of therapeutic VR. This study in people with chronic low back pain (cLBP) evaluated a therapeutic VR intervention called RelieVRx (formerly known as EaseVRx), which consists of multiple pain education, relaxation, biofeedback, mindfulness, and breathing modules. The authors—all being (current or former) employees or advisors of the company that developed this RelieVRx intervention (AppliedVR)—propose that this intervention ‘can target brain regions that are implicated in cLBP.’ The Maddox et al^4^ trial differs from most of the previous therapeutic VR trials by its (a) large sample size of 1067 participants; (b) placebo-controlled design with a sham VR intervention (ie, 2D nature images on VR), which made it possible to control for the potentially large placebo effect of VR; and (c) blinding of both participants and research team members. Their fourth and perhaps most valuable strength is the very thorough and applaudable development process, which started with a pilot study,^5^ followed by a first RCT in 188 participants with cLBP from which outcomes were reported at 8 weeks^6^ and 6,^7^ 18,^8^ and 24 months follow-up,^9^ and now this second larger RCT from Maddox et al^4^ that is published in the current edition of Mayo Clinic Proceeding. Maddox et al^4^ reports that RelieVRx was more effective than sham VR. In this editorial; however, we would like to nuance the at first sight very positive and promising study results and offer possibilities to enhance the effectiveness of therapeutic VR interventions, such as RelieVRx.

Maddox et al^4^ summarize their study findings in the abstract by stating that ‘RelieVRx was superior to sham….’ and ‘…VR program was found to impart clinically meaningful improvements above a strong active control comparison…’ Such a study result in this complex patient group of people with cLBP would be quite remarkable. Indeed, at first sight, the effects of RelieVRx at 8 weeks follow-up appear to be remarkably high: a large standardized within-group effect size of 1.0 for pain intensity and an average reduction in pain intensity of 2 points on a 0-10 scale in the intervention arm. On contrary, standardized between-group effect sizes (ie, additional effect above the effect of sham VR) were substantially smaller and therefore quite less remarkable: 0.24 for primary outcome pain intensity, and even smaller effect sizes for anxiety (0.02), physical function (0.12), depression (0.14), and sleep (0.23). Moreover, 47% of the participants receiving RelieVRx reached a clinically relevant 30% reduction in pain intensity, which means that in 53% this has not been reached. Even more striking, 37% (so only 10%-point less) of the participants receiving sham VR reached this clinically relevant reduction as well. The marginal difference between the experimental and control arms can at least partly be attributed to the sham VR intervention—even while it only consisted of 2D nature images—still possessed important therapeutic factors, such as distraction and relaxation, so was not entirely therapeutic-free, as the authors acknowledge themselves. Therefore, the contrast between interventions is possibly too small to properly evaluate the effectiveness of therapeutic VR. Besides that the effects appear to be less clinically relevant than reported, it should also be noted that outcomes were only reported at 8-weeks follow-up. This means that before we can make a definite judgment on the added value of RelieVRx, we need to wait for the long-term results. Although the previous version of RelieVRx did show similar (but again, small) long-term effects,^7^, ^8^, ^9^ this needs to be replicated in this new Maddox et al^4^ trial. Because of this, we consider the author’s conclusions being too positive and premature. This is undesirable, as this can easily mislead clinicians reading this paper, resulting in unrealistic expectations of the intervention.^10^ We would have preferred this study to be interpreted and presented in a more conservative or at least balanced way.

On contrary, we can still be pleased with RelieVRx being available on the market. This therapeutic VR intervention is a low-risk, accessible and US Food and Drug Administration-approved treatment option for the very difficult patient group of cLBP, in which even small effects can be of value to the patient. Another positive point of view is that we see possibilities to optimize the small effects of this VR intervention. First, Maddox et al^4^ included a heterogeneous cLBP group, whereas their intervention specifically targets patients having problems in pain coping, sleep, distress, anxiety, or depression. Their heterogeneous patient selection is particularly surprising as the authors explained that a large sample of diverse participants with a range of clinical severity and depressive symptoms to better represent real-world patients was one of their reasons to conduct this larger trial at the first place. We assume that in a more targeted patient group, the effects of RelieVRx might be larger. Second, the intervention itself may leave more room for improvement. RelieVRx seems to be primarily aimed at pain education and relaxation, whereas based on the importance of physical activity in cLBP, the addition of physical VR exercises could possibly increase the effectiveness of RelieVRx. Moreover, RelieVRx seems to rely primarily on the working mechanism of distraction, whereas other working mechanisms, such as graded exposure and adherence-related mechanisms^1^, ^2^, ^3^ seem to be ignored. Third, RelieVRx was applied as an in-home (stand-alone) intervention, thereby fully depending on the participant’s discipline and motivation. It seems plausible that when VR is integrated into a treatment in which a clinician (eg, physiotherapist) monitors and facilitates the usage of VR, larger effects can possibly be expected.

We would like to conclude this editorial by stating that therapeutic VR interventions, such as RelieVRx have potential in complex patient groups such as cLBP but does not (yet) seem to be as effective as the authors present. The small effects of RelieVRx could possibly be enhanced and become clinically relevant when provided to a more targeted patient group (such as patients having inadequate pain coping or distress), eventually supplemented by physical VR exercises and offered and monitored by a clinician.

## Potential Competing Interests

The authors declare no conflict of interest.
